# Audit on nursing notes in a psychiatry in-patient setting

**DOI:** 10.1192/bjo.2021.511

**Published:** 2021-06-18

**Authors:** Kavinda Gunathillaka, Mariam Timbo, Stephen Ginn

**Affiliations:** Camden and Islington NHS Foundation Trust

## Abstract

**Aims:**

We aimed to assess the accessibility and informativeness of the content of daily nursing notes through an audit, and improve deficiencies identified.

**Background:**

Nursing notes are an important source of observation findings, of in-ward psychiatry patients.

There can be variations in the quality of the notes as well as information contained within.

A basic level of clarity and information within all notes will be helpful in using these to inform the management of patients.

**Method:**

An audit was carried-out in a ward treating working-age patients for psychiatric illnesses.

Setting standards - standard required of a daily progress note was decided after discussion in multi-disciplinary team meeting (MDT). Clear language and information on; mental-state, medication, meals, physical health, personal care, activities, risks and use of leave, were identified as requirements.

Retrospective audit - First audit cycle was carried-out by assessing the notes two weeks retrospectively. The assessment instrument used a qualitative measurement of the readability of the notes as well as quantitative assessment of the contents.

Intervention - The standards set during the MDT, as well as a suggested format for recording notes, were communicated to the staff through email. Follow-up meetings with individual staff members and MDT, to evaluate staff satisfaction and new suggestions to improve the format were held. Difficulties staff encountered when implementing the format were discussed and resolved.

Second audit cycle - Following implementation of the intervention, the notes were again assessed using the same instrument.

**Conclusion:**

Difficulty in accessing information from the notes was noted in the first audit cycle. The average score for accessibility of information when scored on Likert scale + 3 to -3, was 1. Use of language scored 2 on average. On the second audit cycle, accessibility had increased to 3 on average while language score remained 2.

Quantitative measurement was done for presence of information on; mental state, medication, meals, physical health, personal care, activities, risks and use of time away from ward. All of these parameters showed an increase in the post-intervention second audit cycle. Information on taking meals, medication, and physical health was present 100% of the time in the second cycle. Most improvement was in information on personal care which showed a five-fold increase, from 17% to 89%

In conclusion, standard for nursing notes arrived via discussion and consensus in MDT, has been successful in improving the accessibility and information within nursing notes.

